# Book Reviews

**Published:** 1897-04

**Authors:** 


### BOOK REVIEWS.

---

MANUAL OF THE PRACTICE OF MEDICINE. By A. A. Stevens, M.D., Instructor in Physical Diagnosis in the University of Pennsylvania; Physician to St. Agnes Hospital, etc. Fourth Edition, Revised and Enlarged. W. B. Saunders, 925 Walnut Street, Philadelphia. Price $2.50.

That this Manual of Practice has well supplied the needs of students (for whom it was prepared) is shown by its having passed rapidly through three editions. This edition contains some important modifications and considerable additions. Many of the articles have been almost entirely rewritten. An appendix has been added dealing with the methods of examination of the blood and of the gastric contents. The book will continue to be of service and convenience to students. For some reason the author still retains the the very obscure and unnecessary poetical selection on the title page.

ELEMENTARY BANDAGING AND SURGICAL DRESSING. With Directions Concerning the Immediate Treatment of Cases of Emergency. By Walter Pye, F. R. C. S., Late Surgeon to St. Mary's Hospital. Seventh Edition. Price 75 cents. W. B. Saunders, 925 Walnut Street, Philadelphia.

This little book describes in a brief and practical manner the application of bandages and splints, the dressing of wounds, burns, scalds, and bedsores, the making of poultices, fomentations, the immediate treatment of fractures, improvised splinting, the arrest of hemorrhage, the first treatment of shock and collapse, restoration from drowning, treatment of poisoning, etc. It is made in convenient size for the pocket.

---

INTERNATIONAL MEDICAL ANNUAL. A Work of Reference for Medical Practitioners. Fifteenth year. Price $2.75. Published by E. B. Treat, 241 West 23d Street, New York.

Practitioners are so familiar with the character and value of the successive editions of this work that a lengthy review or description of it is unnecessary. The present edition (1897) is of the same style as its predecessors, presenting in compact space the important and essential features of medical and surgical progress within the year. It is equally deserving of the general favor and patronage which former issues have received.

---

INEBRIETY. Its Source, Prevention and Cure. By Charles F. Palmer. Published by the F. H. Revell Company, New York. Pp. 108. Price 50 cents.

This little book is a moral discussion of inebriety in its various aspects and relations. The author writes well, and from his point of view, has presented this subject in an attractive and interesting manner.

